# Ultra-weak photon emission from DNA

**DOI:** 10.1038/s41598-024-80469-0

**Published:** 2024-11-21

**Authors:** Mariusz Pietruszka, Marek Marzec

**Affiliations:** Faculty of Natural Sciences, Institute of Biology, Biotechnology, and Environmental Protection, University of Silesia, Katowice, 40-032 Poland

**Keywords:** Barley, Dynamic entropy, Hurst exponent, Interference, Light quantum, pH, Photovoltaic current, Time series, Biophysics, Genetics, Plant sciences, Optics and photonics, Physics

## Abstract

It is conventionally believed that macromolecules found in living cells, including DNA, RNA, and proteins, do not exhibit inherent light emission. However, recent studies have challenged this concept by demonstrating spontaneous light emission from nucleic acids under certain conditions and physiological temperatures. By noninvasive monitoring of barley genomic DNA and advanced statistical physics analyses, temperature-induced dynamic entropy fluctuations and fractal dimension oscillations were identified at a key organizational threshold. The study revealed evidence for non-equilibrium phase transitions, a noticeable photovoltaic current jump at zero bias voltage, and a proportional increase (scaling) of the photoinduced current corresponding to increasing amounts of DNA. In addition, we estimated DNA’s energy production rate at criticality and introduced an interferometer using coherent light emissions from the DNA-water interface. These findings suggest that DNA is a major source of ultraweak photon emission in biological systems.

## Introduction

The phenomenon of quantum dynamics plays a fundamental role in the behavior of large-scale systems, including crystals, ferromagnets, and superconductors, where an ordered state is observed^1^. In contrast, biological systems exhibit a unique form of order arising dynamically from the interactions of quantum-level components. This study reveals a macroscopic coherent state characterized by a singular attractor in the system’s phase space, coinciding with a significant reduction in dynamic entropy. Additionally, nonlinear complexity metrics demonstrate distinct extremes at critical points, further supporting this emergent coherent state. The molecular mechanisms regulating optimal life-sustaining temperatures corresponding to homeostasis are connected with spontaneous electric currents, specifically charged avalanches peaking at a critical temperature ($\:{T}_{c}$); The conductivity peak at a critical point externally manifests the collective behavior of DNA during non-equilibrium phase transitions. Our recent studies have challenged the belief that macromolecules in living cells, such as DNA, RNA, and proteins, do not emit light. DNA has been found to emit light under specific conditions and at physiological temperatures. This discovery suggests DNA is a significant source of ultraweak photon emission in biological systems.

### Analysis of temperature and entropy fluctuations in barley genomic DNA: Maxwell Demon vs. Majorana Angel

Maxwell demon is a thought experiment proposed by physicist James Clerk Maxwell in the 19th century. This concept questions the second law of thermodynamics, which states that entropy in a closed system tends to increase over time. Maxwell demon involves a hypothetical creature or device that can sort molecules based on velocities. By allowing fast-moving molecules to pass through a small opening while blocking slow-moving molecules, the demon would create a region in the system with higher kinetic energy and lower entropy, which contradicts the second law. This thought experiment raises questions about the relationship between information and entropy. The discussion about the nature of the second law and information’s role in thermodynamics has been sparked. However, the demon violates the laws of thermodynamics, as is currently understood, because it requires energy input to perform its sorting tasks. The solution to this problem refers to Landauer’s principle^2^ which links information theory with statistical physics. This work explores the Maxwell demon problem in a phase transition^3,4^, where charge carriers change, resulting in a global reduction in dynamic entropy. At a specific (critical) temperature, it is supposed that a phase change occurs in which single charge carriers (+) partially become (like in the two-fluid model of superconductivity) charge couples (2+) that move in opposite directions and have different spins^5^; dissipation-less pairs overtake the role of “fast” particles in the Maxwell model, Bogoliubov quasiparticles of a two-fluid model of superconductivity. However, even a small temperature change can disrupt this unstable state, causing entropy oscillations. An important concept of theoretical physics, Majorana particles, fermions with the unique property of self-annihilation, are also discussed. Majorana particles, though theoretical, hold significant implications for quantum computing and may be linked to the Dirac-delta-type peaks observed in DNA at critical temperatures^6–8^, suggesting a connection to *dynamic* quasiparticles of the Majorana type. This is possible because a quasiparticle ($\:{c}_{\varvec{k},\sigma\:}{c}_{-\varvec{k},-\sigma\:}$) in a superconductor can act as a particle-antiparticle couple^5^. However, this work presents the concepts of Maxwell’s demon and Majorana-type quasiparticle for interpretative purposes only.

DNA is primarily known for its biological function as the carrier of genetic information. Its physical and chemical characteristics also make it a potential material for various nanotechnology, bioengineering, and materials science applications. DNA’s ability to self-assemble into predictable, programmable structures makes it the candidate for use in nanotechnology. DNA can form complex three-dimensional structures through complementary base-pairing. For example, short DNA strands can be designed to bind together in specific patterns, allowing the construction of nano-sized scaffolds and frameworks. This can be used to build nanostructures for various purposes, such as drug delivery, molecular electronics, or sensors^9^. On the other hand, DNA origami refers to folding a long strand of DNA into a specific shape or structure, which creates intricate 2D and 3D structures, such as nanocages, nanorobots, and molecular machines^10^. Another property of DNA is the high density of information storage – based on its physical dimensions, DNA has a theoretical data density of 6 bits for every 1 nm of polymer, or ∼4.5 × 10^7^ GB/g^11,12^. Hence, researching the properties of DNA molecules is crucial for biological issues and their use in various industries.

Our approach, experiments, and previously proposed concepts (such as Bose-Einstein condensate, Cooper pair, Josephson-like junction, etc.)^5^ partially align with the recent paper describing a potential quantum computer based on the DNA^13^.

## Results and discussion

The ELoPvC technique, as described by Pietruszka et al. in 2018 ^14^, has been used to measure the electromotive force (EMF) in various plant and animal samples, such as pollen grains^15^, human blood^16^, and double-stranded fragments of coding DNA with a specific sequence and length of 1100 bp^5^. Here, we tested whether the DNA voltage could be measured through EMF using the genomic DNA of barley at a concentration of 100 ng/µl. It is important to note the barley genome is estimated to be 5.3 Gbp, with coding sequences constituting no more than 10% ^17^. It means that the results obtained are not specific to a particular DNA sequence but rather stem from the physicochemical properties of the DNA molecule itself.

**Fig. 1:**
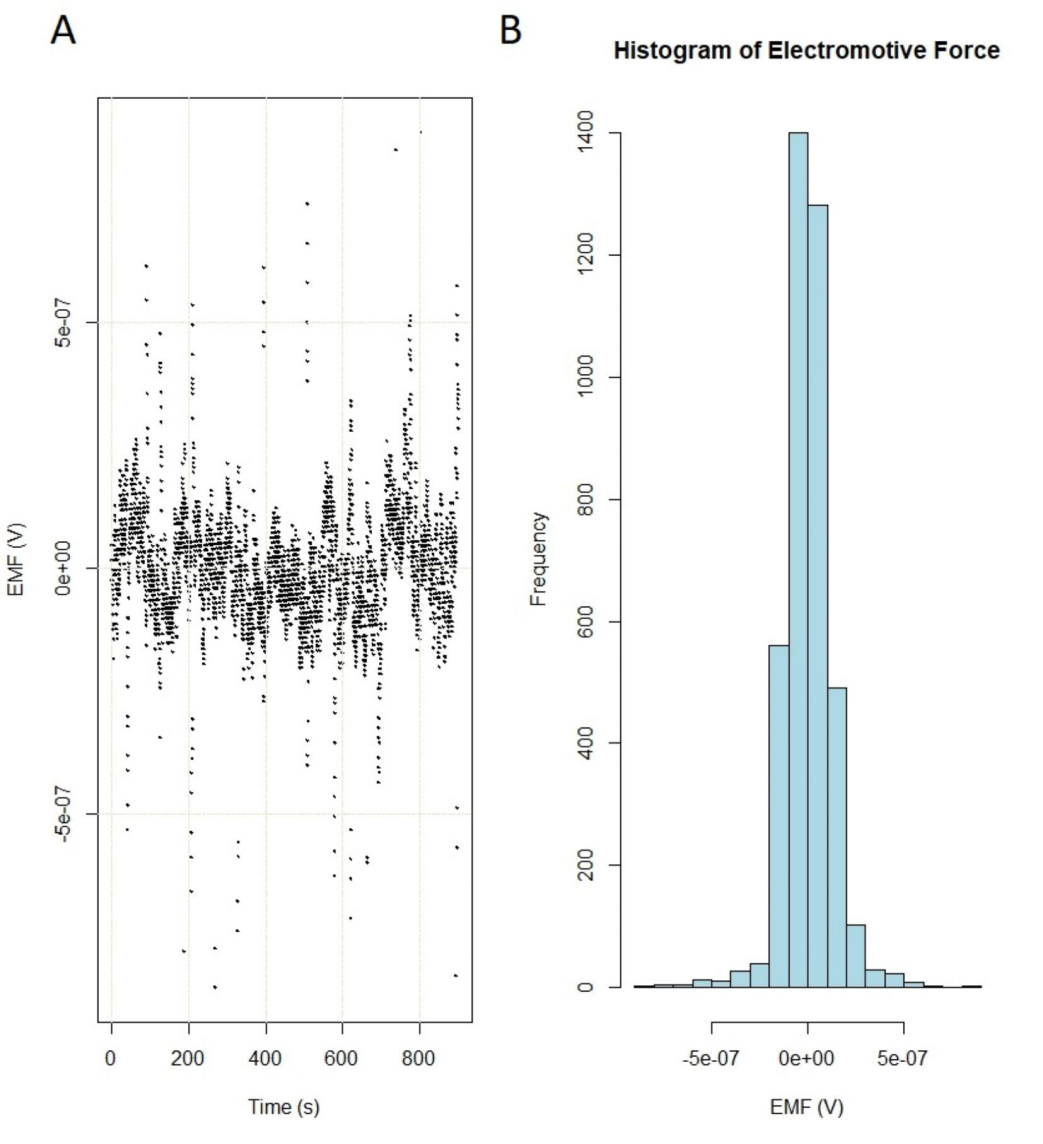
The electromotive force (EMF) of barley genomic DNA at 20.7(1)°C and pH 8.3, i.e., beyond a phase transition. (**A**) Detrended original data as a function of time and (**B**) histogram (Gauss) of the EMF beyond a phase transition ($\:{T\ne\:T}_{c}$).

**Fig. 2:**
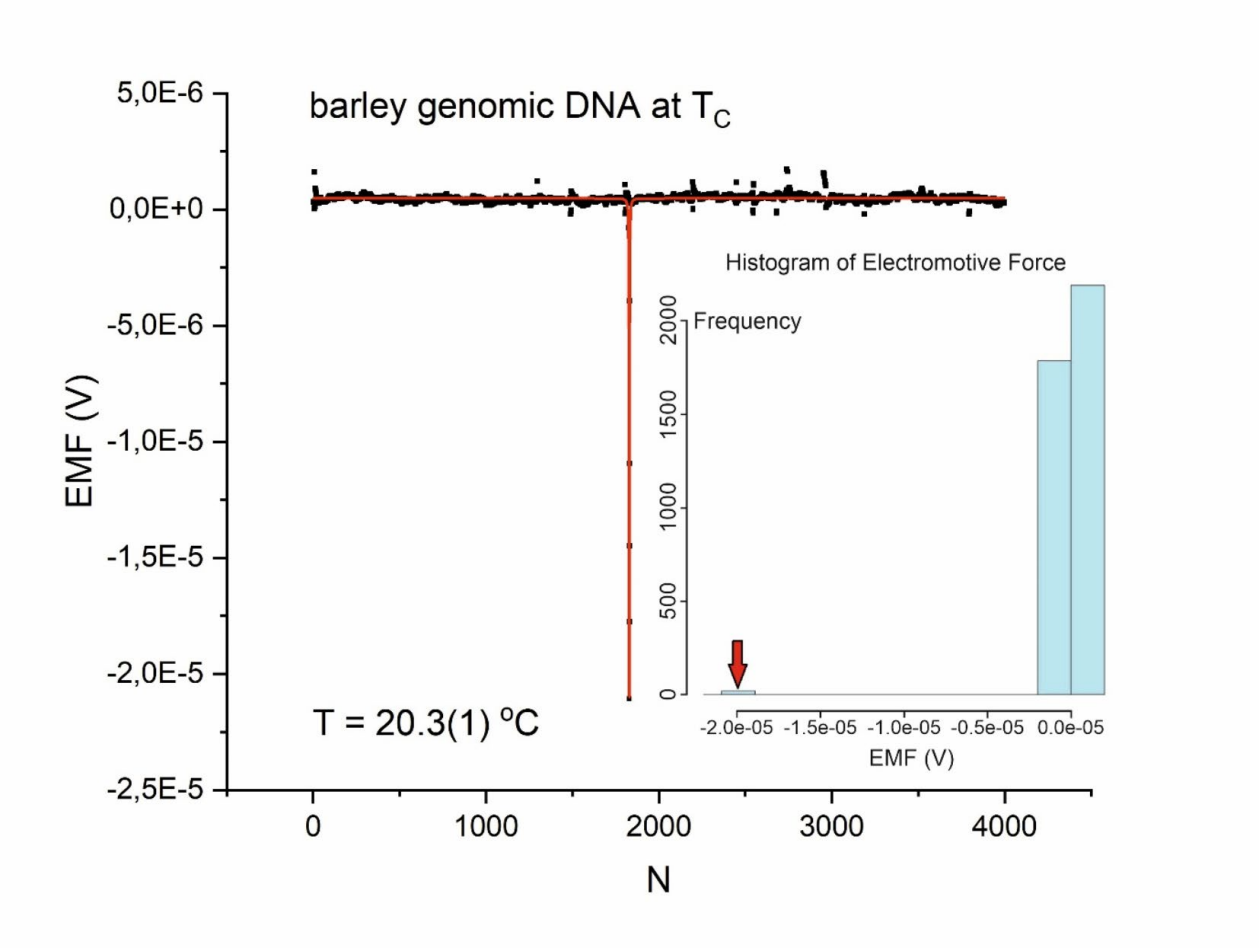
The electromotive force (EMF) of barley genomic DNA at 20.3(1)°C and pH 8.3 was interpolated with the Lorentz resonance curve (R^2^ = 0.81426)—Inset Histogram of the EMF at the critical point $\:{T=T}_{c}$. $\:N$ denotes the measurement number; the sampling frequency was 4.1 Hz. The mean bias for the first 501 data points is 4.0E-7 V with a standard deviation of $\:\sigma\:=$ 1.0E-7 V, resulting in a narrow peak of approximately$\:\:800\times\:\sigma\:$ intensity (amplitude) at $\:{T}_{c}$ (see also Figure S1). The peak can be associated with radiative decay – the emission of photons from excited states.

At a temperature of 20.7(1)°C and pH 8.3, barley genomic DNA suspended in 50 µl of TE buffer (10 mM Tris-HCl, 1 mM EDTA) produced white/pink noise (a) with Gaussian characteristics (b), as shown in Fig. 1. However, at a specific temperature of 20.3(1)°C and pH 8.3, an EMF (voltage) peak, a sign of interaction, appeared that was not present in the control (genomic DNA in 50 µl of TE beyond $\:{T}_{c}$) sample (Fig. 2). A sharp resonance peak centered at 20.3(1)°C occurred, which can be interpreted as a symmetry breakdown in the system. Figure S1 in the Supplementary Information shows the result with a better resolution.

### Entropy oscillations – a Maxwell demon?

**Fig. 3:**
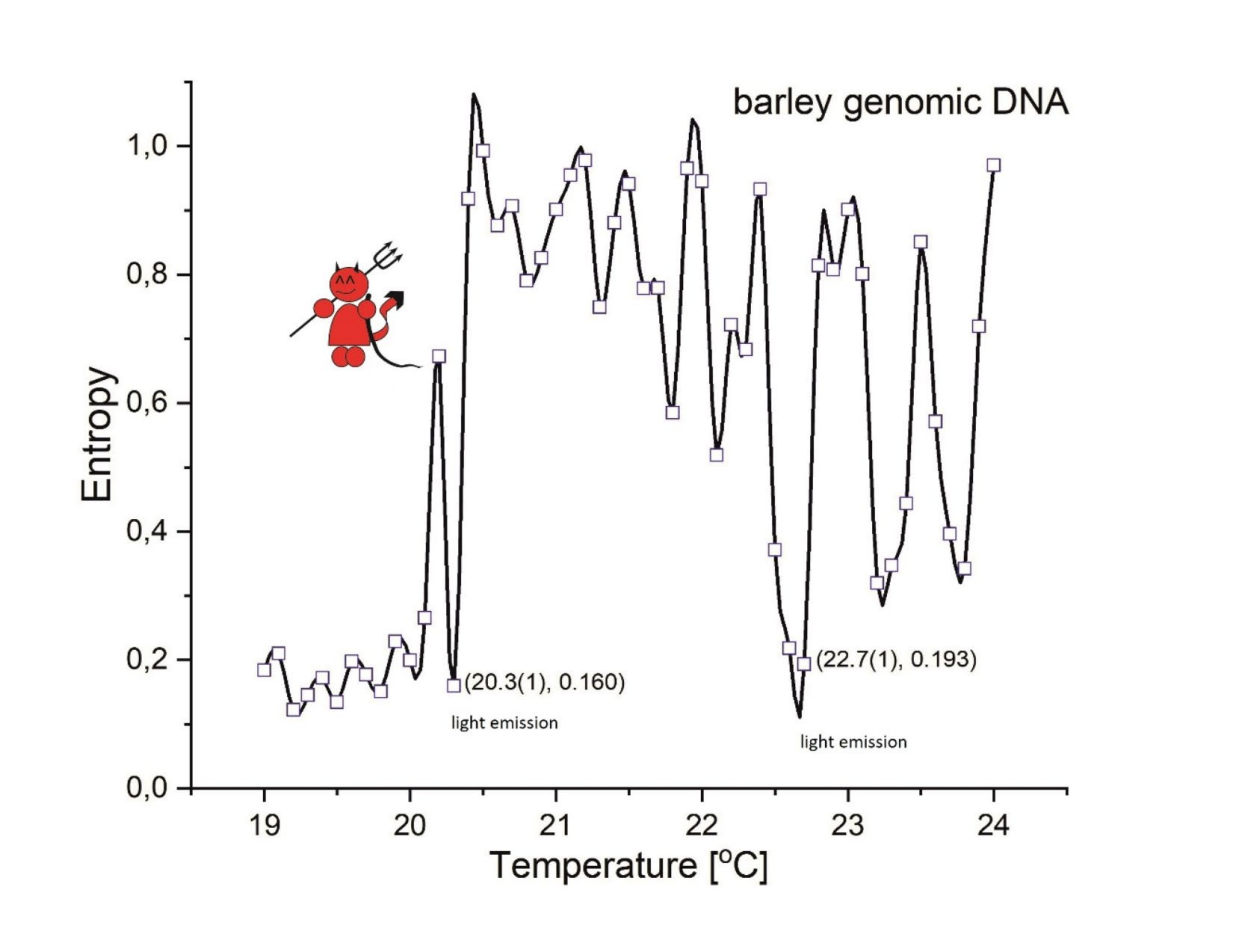
The calculated dynamic entropy of barley genomic DNA at pH 8.3 as a function of temperature (double-drop experiment, bridge closed). The plot shows the results obtained from 4000 real numbers in each experimental time series (and the corresponding temperature) using an approximated entropy procedure in the R programming language. The plot is interpolated with cubic splines and highlights a low-entropy (oscillations) area on the left, a high-entropy void in the middle, and (developing) high-amplitude entropy oscillations to the right. The two main low-entropy peaks are labeled with their respective coordinates. The plot is based on 200,000 measurements at 4.1 Hz sampling; the temperature resolution was 0.1 °C. A Maxwell demon that uses information about a system to reduce its entropy to gain work is symbolically presented in the plot. The light emission is indicated at the pronounced local minima following Fig. 2, and earlier observations^5^.

The opportunity for entropy oscillations around a stationary state in linear and nonlinear processes was theoretically shown in Ref.^18^. This prediction provided the first experimental evidence in the present work when the water (reservoir, or solvent) immersed genomic DNA (sample) was used (Fig. 3). After some number crunching calculations (R), we observe low entropy (stationary state) behavior at 19–20 °C, where increasing disorder prevails at ΔS > 0, a high entropy occurs at 20.5–22.5 °C, and pronounced low entropy peaks occur (indicated in the plot) above. The entropy oscillation period around a stationary state at low temperatures equals 0.86173E-5 eV. In the author’s opinion^18^, entropy oscillations should occur as alternating self-organization processes and disorganization. These oscillations can arise after the system reaches the critical level of organization (Fig. 2, ibid.). Pietruszka’s 2021 study^7^ already revealed (dynamic) entropy^19^ oscillations. In study^7^, the minimum dynamic entropy was approximately 0.0001 eV wide, featuring a dynamic “Majorana-type” quasiparticle peak at its center. These results can be compared with those presented in Fig. 3 and Figure S1. Moreover, as predicted by Shapovalov^18^, the processes of ordering and self-organization should prevail below the critical level where ΔS < 0, and processes of disorganization, where ΔS > 0, should have a higher critical level. This phenomenon, however, in the temperature domain is visible in Fig. 3, where the “great void space” is limited by two local entropy minima, see also^5^. The small-amplitude but regular (periodic) oscillations to the left of the first entropy minimum develop into large-amplitude oscillations at higher temperatures, as depicted in Fig. 2 in Ref.^18^. The described processes coincide with the least entropy production theorem^20^ in DNA systems at critical points.

### DNA: light within?

According to our research, barley genomic DNA dissolved in TEs can produce a small amount of energy. This energy is generated by a spontaneously polarized peak voltage (EMF) of 5 × 10^− 7^ V and a spontaneous peak current of 5 × 10^− 8^ A at a critical temperature of approximately 20.3(1)°C (the two independent experiments yielded measurements for both values). The power produced by this current is approximately 2.5 × 10^–14^ W. Hence, the DNA energy production rate can be estimated (for a sample containing 100 ng/µl genomic DNA) to be approximately 25 mJ/g/second. From the formula $\:\lambda\:=\frac{hc}{E}$, the wavelength corresponding to 25 mJ is approximately 79.5 nanometers. It falls in the ultraviolet range at 3.75 × 10^15^ Hz of the electromagnetic spectrum. On the other hand, assuming that DNA excitations occur through collisions with environmental particles, the conversion of thermal energy ($\:E=\frac{1}{2}kT$) into radiant energy ($\:E=\frac{hc}{\lambda\:}$) gives the value of $\:\lambda\:$ as $\:2\frac{hc}{kT}=96$ µm for $\:T=300$ K (room temperature).

More than fifty years ago, Herbert Fröhlich presented a concept of long-range coherence in biological systems^21^, see also Ref.^22^. and papers cited therein. He proposed the Bose-like condensation in non-equilibrium conditions. Synchronous, large-scale collective Fröhlich oscillations imply intercellular photon emissions, constituting a non-chemical and non-thermal interaction. These oscillations could be revealed by detecting GHz or THz radiation emissions^23^. A respective UV-light spectrometer (or microwave cavity) should measure the emitted radiation wavelength to allow the number of photons calculation. We suggest that DNA molecule oscillations release energy, achieving the coherent oscillation at criticality. Moreover, the spectroscopic properties of the Fröhlich condensate, are especially revealed by the narrow linewidth (compare with Fig. 2). This gives the condensate *long-lived coherence* and collective motion^24^; see the steps in Fig. 10.

### DNA-induced photovoltaic current

**Fig. 4:**
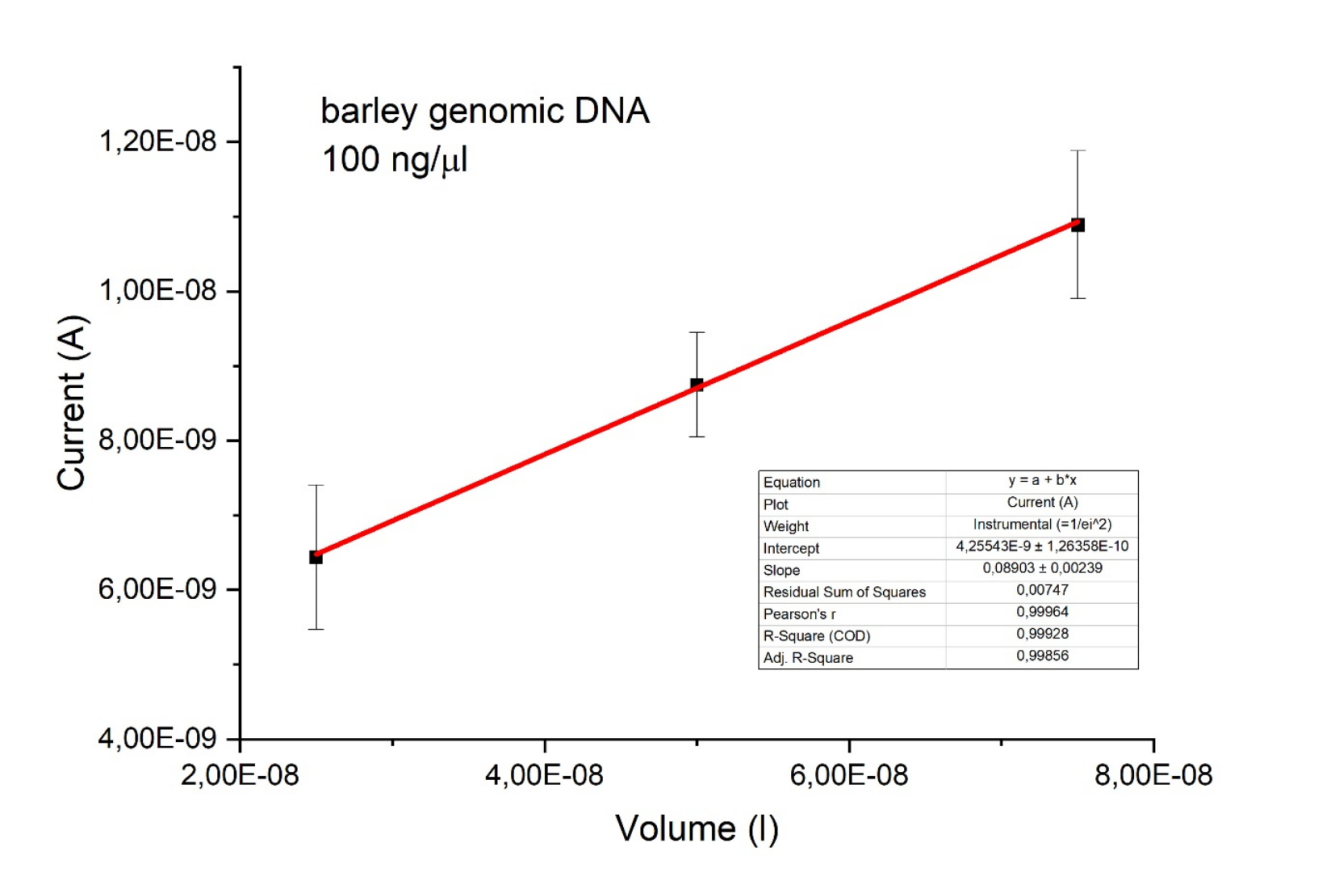
The relationship (linear scaling) between the DNA-light-induced photovoltaic current and the volume of the barley genomic DNA samples. The line of best fit has an adjusted R-squared value of 0.99856. The equation for the (current) line is $\:I={I}_{0}+b{V}_{DNA}$, where $\:{I}_{0}$ is the intercept and $\:b$ is the slope, which is approximately 9%.

**Fig. 5:**
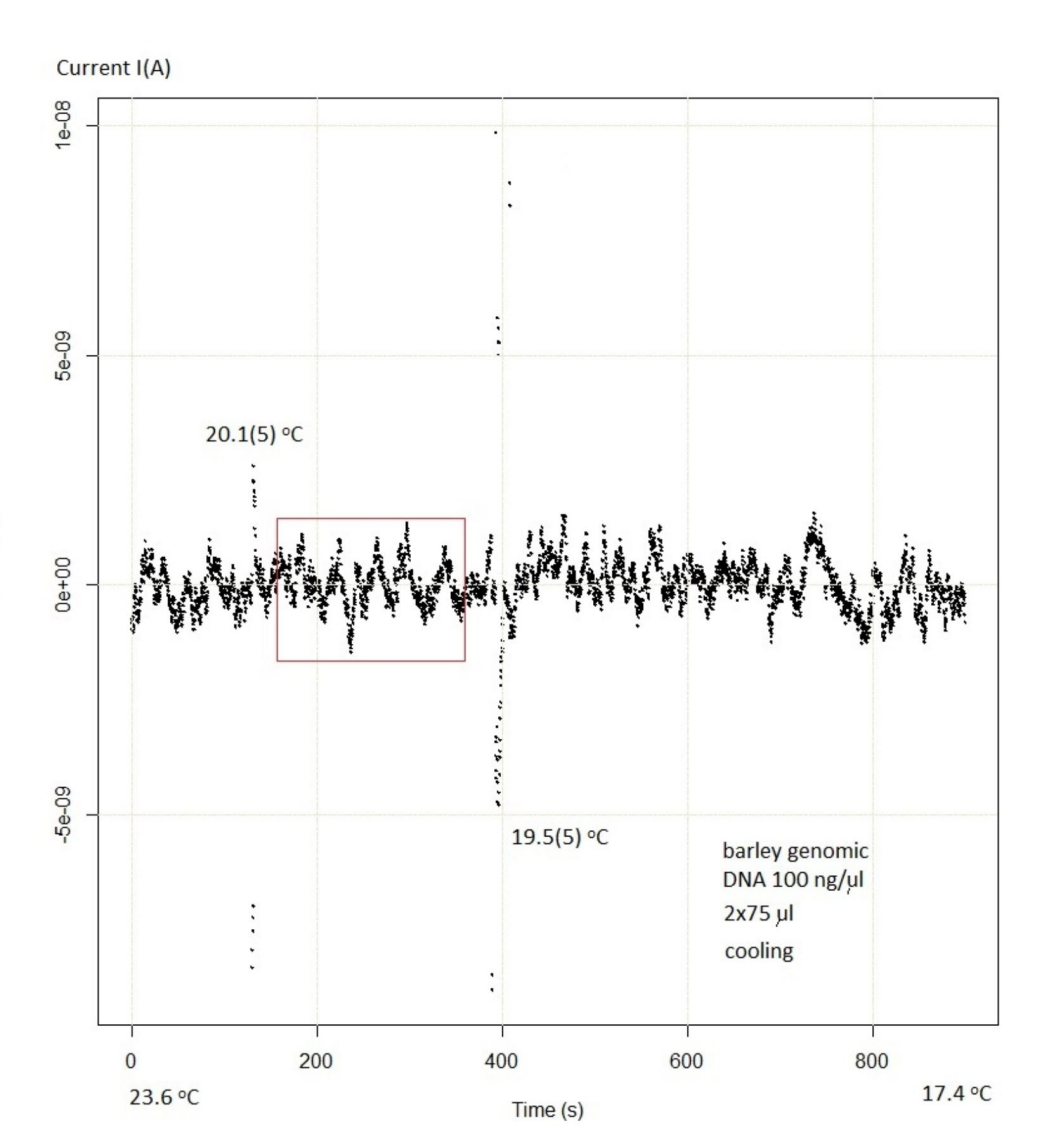
Coherent state, (expectation value) of the photon-induced current, as a function of time showing the quantum fluctuations (frame). The fluctuations (corresponding to the* line thickness*) are always the same, such that the field is as close to a classical field as possible for any quantum state (compare with Fig. 3.3 in Ref.^32^). A light field has its highest degree of coherence (laser action) when its corresponding interference pattern has maximum contrast on the screen. The interferometric (wavy) pattern was obtained in the double-drop (bridge open) experiment (compare with Fig. 4 in Ref.^23^). Upon cooling with cold water to the physiological temperature range, the genomic DNA of barley induces a photovoltaic current (detrended data) that exhibits (coherent) sinusoid-like characteristics (frame) and enhanced oscillations (peaks) at critical temperatures (see also Supplementary Figure S4 and Movie 1). The signal is smeared out (fuzzy) at 17.4 ^o^C. The “coherence time” (frame) equals ~ 25 s and depends of the rate of temperature change.

Figure 4 compares the volume of the barley genomic DNA samples and the induced photovoltaic current. The linear fit of the data showed that the adjusted determination coefficient R^2^ was 0.99856. The linear equation $\:I={I}_{0}+b{V}_{DNA}$ represents the empirical relationship between the current and the light-emitting DNA sample volume, where $\:{I}_{0}$ represents the intercept and the slope $\:b$ is approximately 0.089(2), equivalent to approximately 9%. Hence, the current is directly proportional to the sample’s number of carriers. This is because the more DNA particles there are, the greater the number of photons emitted. Linearity also indicates that there is no interaction between the individual copies of DNA molecules allowing for the creation of a great statistical ensemble (say, one million copies, a regular amount in the Eppendorf tube) in future applications, where the Hamiltonian reads: $\:H\leftarrow\:H-\mu\:N$ (where $\:\mu\:$ is the chemical potential). The fluctuating “electromagnetic vacuum” in Fig. 2 or Fig. 5 can produce a double (up/down) peak, at either 20.1(5) °C or 19.5(5) °C, respectively, which may be interpreted as the formation of a quasiparticle (particle-antiparticle pair) followed by annihilation and light emission. [Note that the data was sampled at about 4 Hz, resulting in sparse points every ¼ s]. Compare with Supplementary Figure S5.

While it has been reported that stochastic fluorescence switching in nucleic acids can occur under visible light illumination^25^, there is no mention in the scientific literature of the natural emission of light (the release of energy, in the form of photon emission) by nucleic acids. Nevertheless, Popp and co-workers have reported biophoton emission or fluorescence from living systems (soybean cells and seedlings)^26^. This could be because energy quanta emission can be detected only under specific conditions. There is an anecdote about a sprinter who only runs a sprint for 10 s, but spends most of his time sitting, walking, or sleeping. This is similar to the behavior of DNA - it glows only for a very short time when the appropriate physical (temperature) and chemical (pH) conditions^5^ are met in the body. It is therefore likely that earlier observations (if any) were not successful; the “runner” (DNA molecule) was most of the time at idle. Moreover, it can be expected the transition to a lower energy state at $\:{T}_{c}$ would emit light quanta: a two-qubit system of two nucleotides with 2^2^ = 4 states can be represented by only two entangled states and the excessive energy must be emitted. Recombination of the electron-hole pair of a quasiparticle in an oscillatory resonant quantum state^13^ would also emit light quanta in the appropriate frequency band, not to mention the Fröhlich condensate with the suggested frequency of 10^11^ Hz. The peak of cosmic microwave background radiation filling the Universe localizes at 160.2 GHz = 1.602 × 10^11^ Hz and can be compared with Fröhlich’s suggestion. Microwave radiation has small energies that are insufficient to ionize atoms. At the highest frequency, the energy per photon is less than 10^− 3^ eV, an energy range (~ 0.0007 eV) we deal with in our studies^5^.

### Biophotons and cellular communication

Biophotons are optical or ultraviolet photons emitted by living cells, different from conventional bioluminescence. Although the exact mechanism of biophoton production is still unclear^27,28^, there is increasing evidence that cells emit these photons. According to Ref.^24^, biophoton streams exhibit *short quasiperiodic bursts*,* similar to those used for binary data transmission over noisy channels*; for visual comparison, please refer to Fig. 2 and Supplementary Figures S6-S7. The situation resembles the challenging information transfer in the “wet and noisy”^29^ environments within biological systems.

### Fluorescence of DNA macromolecules

Living cells contain molecules that emit fluorescence when excited by visible light. This fluorescence can be captured without the use of fluorescent stains. Even DNA, usually not fluorescent, can occasionally emit light and remain non-fluorescent for long periods. The natural fluorescence from these molecules can be even brighter than that achieved with fluorescent labels, which makes it ideal for imaging without the toxicity associated with staining. In summary, if DNA naturally emits light at criticality, it could present exciting possibilities for label-free, super-resolution nanoscopic imaging for a deeper understanding of biological processes.

### Is photon emission from hydrogen bond resonance possible?

Yes, photon emission linked to resonance in the hydrogen bonds of, e.g., A-T base pairs could be possible under certain critical conditions (such as critical temperature or pH), particularly if those conditions induce changes in the protonation states, electron density distribution, or the molecular structure of the base pairs. These changes could lead to excited states in the base pairs, and the subsequent return to lower-energy states could result in the emission of photons.

### DNA-emitted light interferometer

Moreover, we observed sinusoid-like oscillations (corresponding to interference fringes) and a current enhancement (resonance peak) at a critical temperature $\:{T}_{c}$ = 20.3(1)°C and pH = 8.3(1) using a simple DNA-emitted light photovoltaic interferometer (Supplementary Figures S3,S4). These preliminary results are shown in Fig. 5. Additionally, Supplementary Figure S4 displays the double-dot configuration of the experiment, and Supplementary Movie 1 shows the current oscillations on a Multimeter display. The comparison of Fig. 5 and Supplementary Figure S5 shows that the results are reproducible, however, the intercalation of a staining substance blurs the purity of the signal, even leading to the vanishing of the peak at $\:{T}_{c}$.

**Fig. 6:**
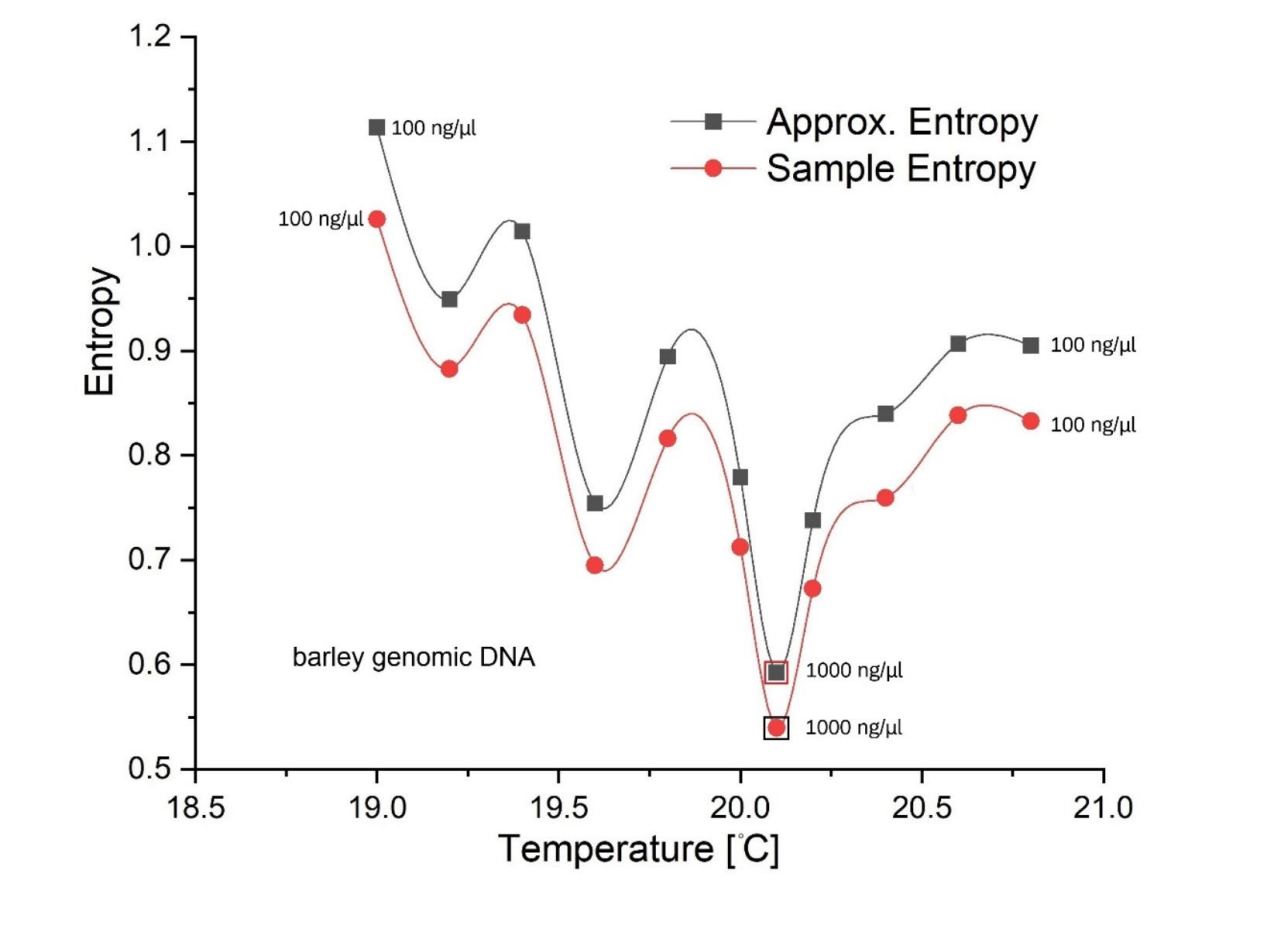
Dynamic entropy of barley genomic DNA was calculated for a double-drop experiment (see Figure S4, bridge open) using approximate and sample entropy procedures in R. The DNA concentration was 100 ng/µl, except for one point (indicated) at a global minimum (1000 ng/µl) where both minima coincide. Cubic splines interpolate data.

In the double-dot experiment (refer to Figure S4), the dynamic entropy of genomic DNA in barley was computed (Fig. 6) using approximate and sample entropy procedures (R). The concentration of DNA was maintained at 100 ng/µl, except for one additional point (from independent experiment) at the global minimum (1000 ng/µl), where minima for both concentrations coincide. Additionally, a current peak at $\:{T}_{c}$ and the steps in the current (with slightly changing temperature or energy), both occurring at zero bias voltage in a double-dot experiment are shown in Supplementary Figures S6 and S7, respectively.

**Fig. 7:**
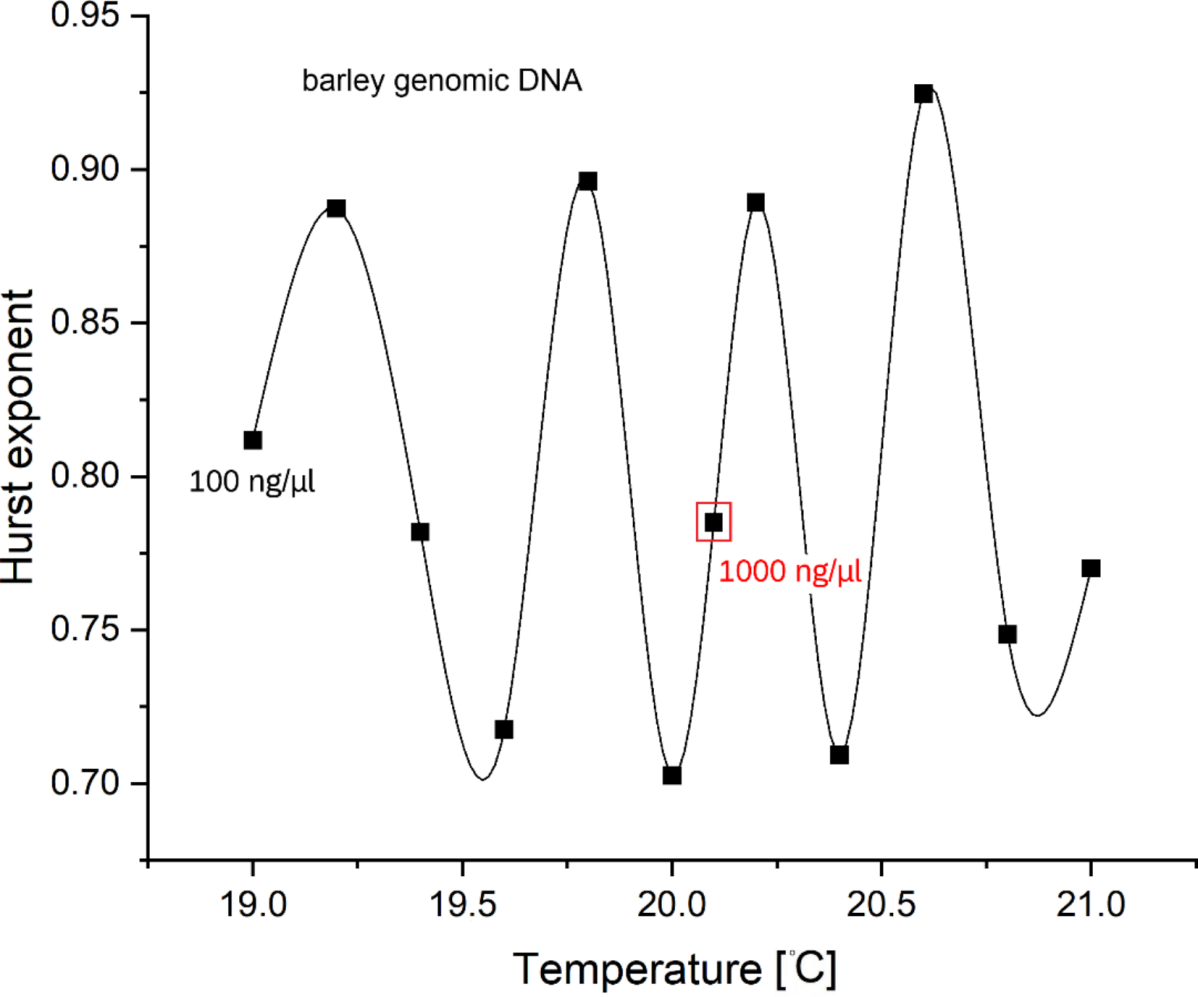
Hurst exponent ($\:H$) against the temperature of barley genomic DNA computed for a double-drop experiment (see Figure S4, bridge open) using R programming language procedure. Through the relation $\:D=2-H$, the plot can be interpreted as temperature-dependent fractal dimension ($\:D$) oscillations. The DNA concentration was 100 ng/µl, except for one additional point (1000 ng/µl, indicated) showing a good fit between neighboring points. Cubic splines interpolate data.

The Hurst exponent of barley genomic DNA was calculated for a double-dot experiment using the R programming language procedure and you can see the results in Fig. 7. Conducted with the bridge open (flooded), a weak contact equivalent to a Josephson junction weak link was established; see Figure S4. The DNA concentration was maintained at 100 ng/µl, except for one additional point having a concentration of 1000 ng/µl. This extra point obtained in the independent experiment was added to demonstrate an (optically) ideal interpolation between the neighboring points. The experiment revealed that barley’s genomic DNA concentration does not affect dynamic entropy or Hurst exponent (or fractal dimension). As stated earlier, it means that there was no interaction between the individual copies of DNA.

**Fig. 8:**
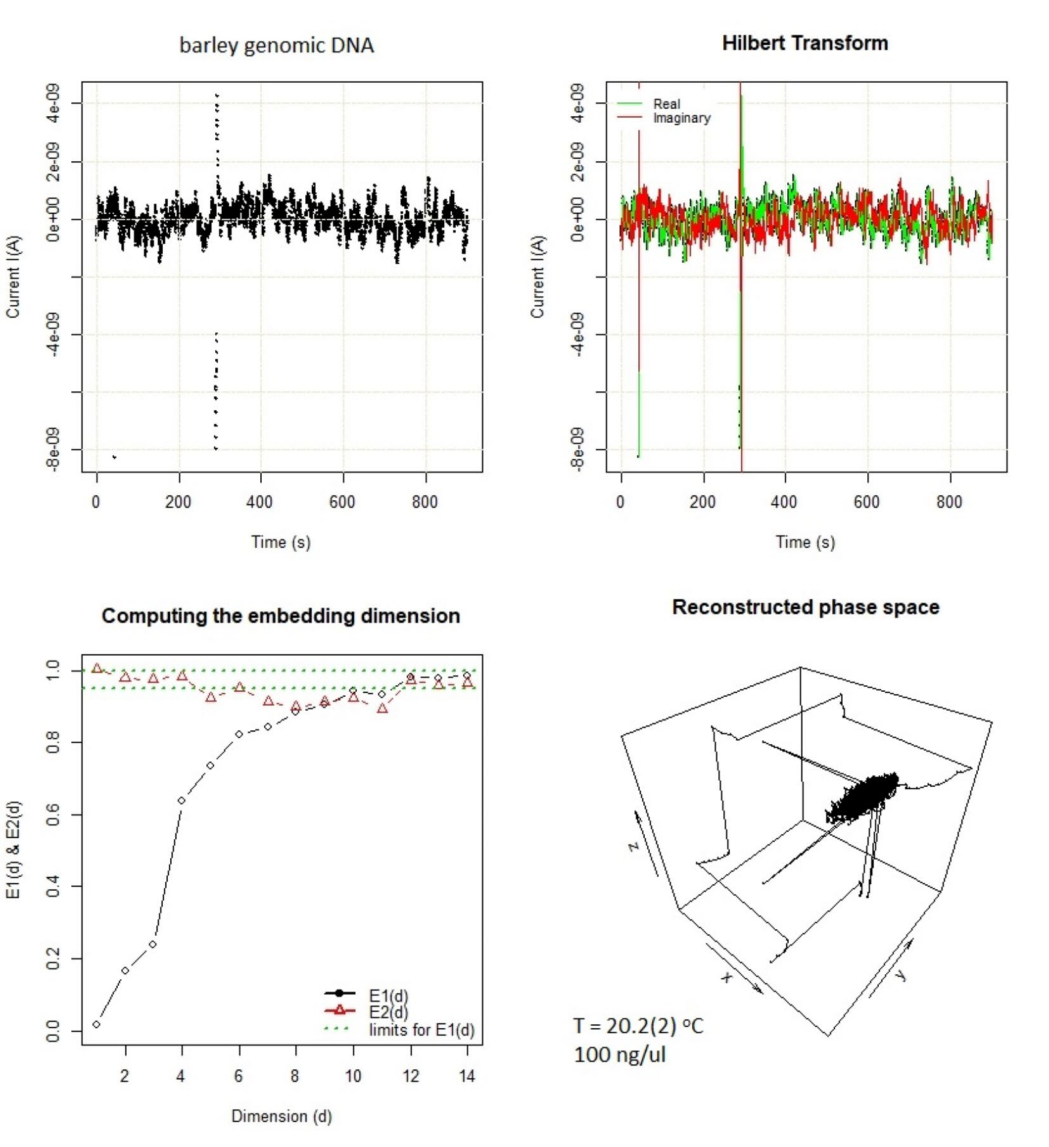
Detrended data of an electrical current time series, the Hilbert transform, computing of the embedding dimension (Takens theorem), and the reconstructed phase space of 100 ng/µl barley genomic DNA.

After removing the trend from a time series of electrical currents, we used Takens’s theorem to calculate the embedding dimension. This allowed us to reconstruct the phase space of barley genomic DNA. Figure 8 shows the reconstructed phase space along with the Hilbert transform. We performed all the calculations in R programming language, using the configuration shown in Figure S4 with the bridge closed. The prominent peaks are identified and reconstructed in the phase space trajectory.

### Magnetic field effect

**Fig. 9:**
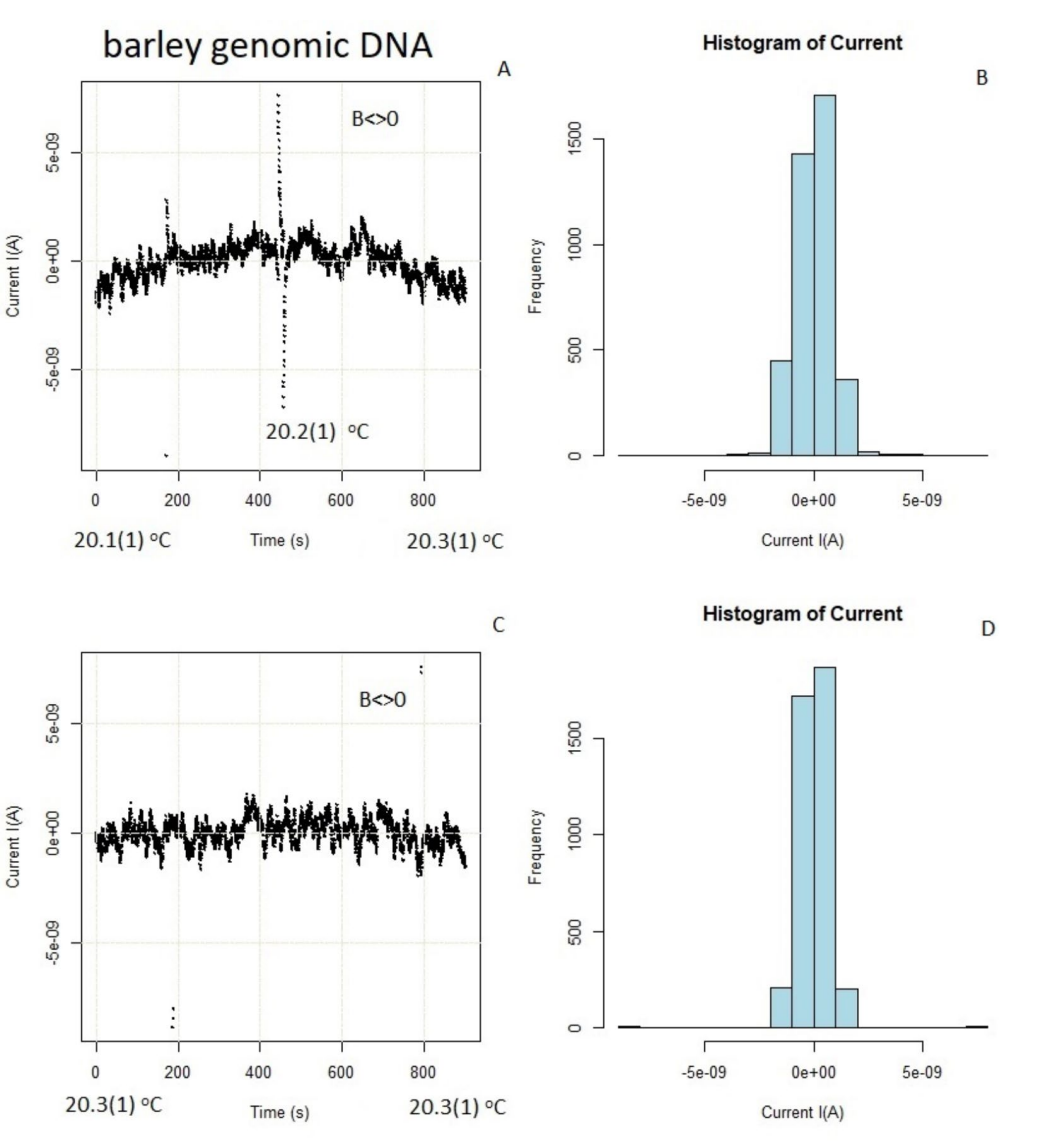
Barley genomic DNA in the presence of a ~ 0.2 T magnetic field. (**A**,**B**) The current as a function of time at about $\:{T}_{c}$ – photon emission/absorption peak is visible. It can be, e.g., explained by the oscillatory resonant quantum state in DNA nucleotides. (**C**, **D**) The current above $\:{T}_{c}$.

When a constant magnetic field with a strength of about 200 mT was applied, it caused changes in the current time series, as demonstrated in Fig. 9, and Figure S8 (also refer to Figure S9 for the neodymium-based instrument). The extended peaks previously observed near the critical temperature disappeared, and highly symmetric two individual points appeared at $\:{T}_{c}$ (which may account for the decay into a particle-antiparticle pair). This suggests that magnetic induction has an influence (through $\:\overrightarrow{A}\bullet\:\overrightarrow{p}$ matrix elements) on the system’s evolution. The emergence of these points can be explained by the presence of quasi-particles, known as “Cooper pairs”^13^, which transform into separate real particles when subjected to a strong magnetic field. However, this observation can also be interpreted as tautomeric mutations^30^, prohibited in a strong magnetic field; as previously mentioned^6–8^, Earth’s magnetic field can assist in preventing tautomeric mutagenesis. On the other hand, examining Figure S8 raises the possibility that living structures can communicate through waves, just as humans have recently started to do for social purposes^31^.

A Van Hove singularity refers to a (Dirac) point in the density of states (DOS) of a crystalline solid where the DOS is non-smooth. In one-dimensional structures like a DNA strand, the DOS - directly proportional to the current - becomes infinite at points where the gradient of energy ($\:\nabla\:E$) becomes zero. Figure S8 shows a photovoltaic current as a function of temperature that can be converted to energy (*E*), causing singularities to occur. These anomalies in spectroscopic measurements are usually attributed to Van Hove singularities. The specific electronic density of states essentially mirrors the processes in DNA (where $\:D\left(\omega\:\right)d\omega\:$ is the number of modes in a range $\:d\omega\:$^21^) when detecting light flashes at the n-p photovoltaic junction. The Hamiltonian (energy function) for the studied system ($\:{H}_{DNA}$) should thus resemble the Hamiltonian of the semiconducting light detector $\:{H}_{detector}$, incorporating the proton-to-electron mass ratio (1836). Consequently, the symbolic mapping $\:{H}_{DNA}\to\:{H}_{detector}$ could offer a new theoretical framework for exploring DNA events using methodologies established in many body solid-state physics. For experimentalists, a simple DNA photovoltaic spectrometer can be developed and proposed. Either way, our results can be inspiring for theoretical and experimental physicists.

### Quantum leaps and the Quantum Nature of Light

**Fig. 10:**
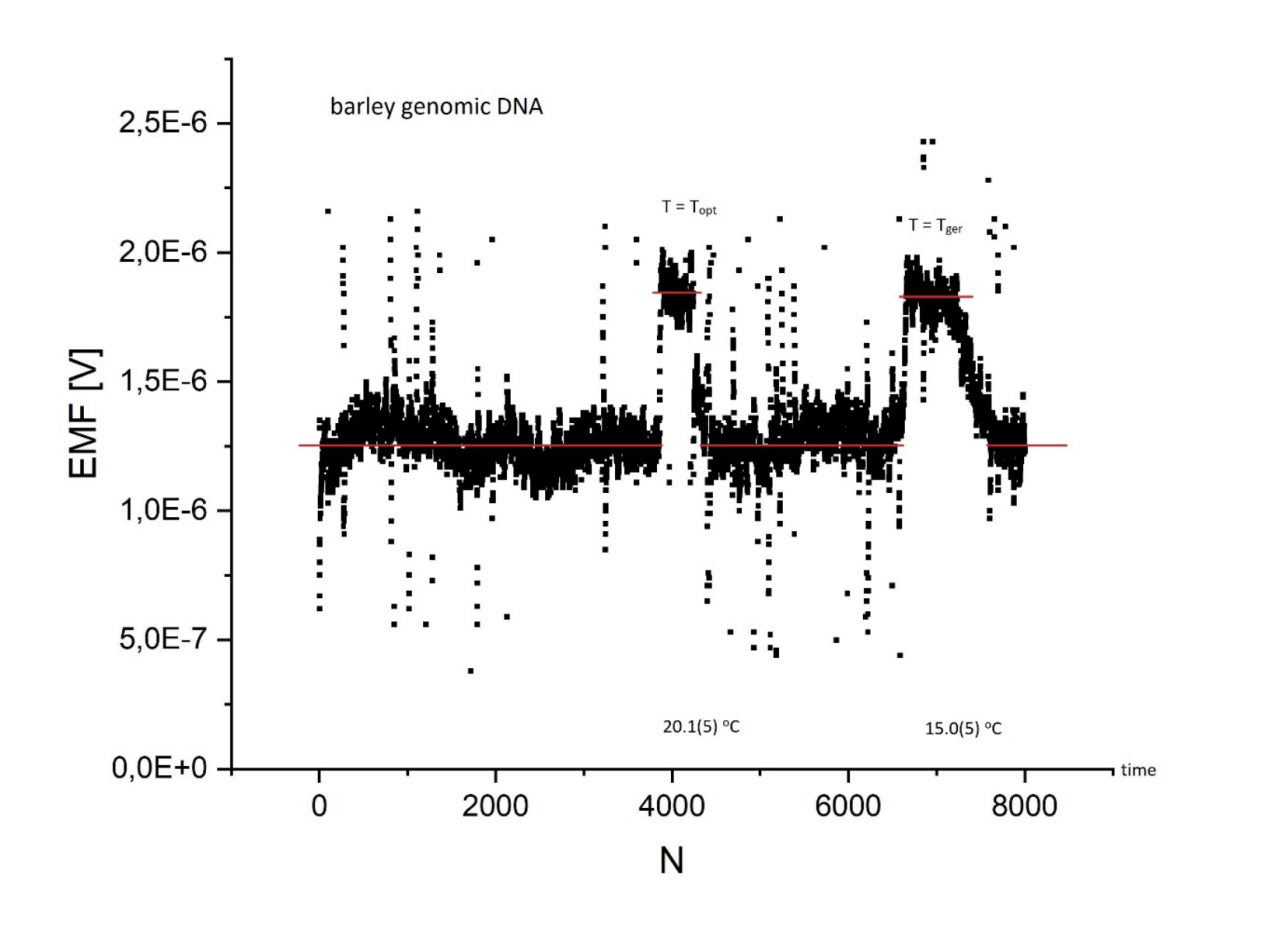
The electromotive force of barley genomic DNA (concentration 100 ng/µl): recording the *spontaneous* light signal (light intensity $\:I\left(t\right)$ as a function of time, see also Fig. 8.2 in Ref.^32^) , showing moments of quantum leaps and long-lived coherence at critical temperatures during cooling (27.5–14.9 °C) with ice water.

All states of light are quantum, and therefore nonclassical because their quantum properties are a consequence of the discrete nature of the photon^32^. Thus, the photovoltaic current seen in Fig. 10, which is proportional to the number of photons as a function of time, is a direct observation of spontaneous light quanta emitted by the DNA molecule. Besides, the graph in Fig. 10 displays quantized jumps (please compare with Fig. 8.2 in Ref.^32^) of the light-emitted signal at critical temperatures; periods in which a high photon counting rate is separated by periods in which this rate is close to zero.

Similar to our previous paper^6^, two characteristic temperatures of barley germination ($\:{T}_{ger}$) and optimum growth ($\:{T}_{opt}$) are also shown. The cardinal temperature for germination is 15 °C, while the optimum temperature for growth is 20 °C, which agrees with Fig. 10. The characteristic temperatures differ from the corresponding specific temperatures for hyacinth or tobacco^6^.

## Conclusions

This interdisciplinary report focuses on studying temperature and entropy variations in the genomic DNA of barley. We conducted noninvasive measurements *via* a semiconductor–electrolyte interface and used statistical physics calculations to reveal nonequilibrium phase transitions occurring in the barley genomic DNA. At a specific temperature of $\:{T}_{c}$ = 20.3(1)°C and pH = 8.3, we observed the following phenomena:

1. A resonance peak in the electromotive force and a significant decline in dynamic entropy.
2. The current peak at zero bias voltage; it can be attributed to the Bogoliubov quasiparticle^5^.

This behavior (collective excitations) could arise from the dynamic interaction^1^ of proton pairs with opposite momentum and spin^33^ or self-organizing^6^ synergetic dissipative structure, the Fröhlich condensate^22^. These pairs carry a charge of q = 2 + under critical conditions in a liquid (water dipole) DNA environment^5,34^. The photovoltaic current induced by light scales linearly with the amount of DNA in a sample, indicating independent but synchronous action of these molecules, at least at low concentrations. Due to higher temperature resolution measurements, dynamic entropy oscillations around a critical level of organization were observed for the first time. After conducting noninvasive experiments, including non-equilibrium thermodynamics and advanced statistical physics methods, we have also integrated well-known concepts from physics and philosophy (see beneath). Furthermore, we have estimated the energy production rate of barley genomic DNA.

A new type of interferometer has also been introduced that uses light emitted by DNA, which is a measuring device based on the principle of wave interference. Interferometers work by superimposing two coherent waves, creating areas of extinction and amplifying vibrations. Accurate measurements can be achieved by observing the interference patterns and making precise calculations. It’s worth noting that for this to work, the light emitted by DNA must be coherent. Life exhibits a distinctive signature of a wavy, cyclic pattern also present in DNA (Fig. 5). This pattern can facilitate critical modes of interaction between different molecules through interference, essential for correlations in a prebiotic world. Proton tunneling (quantum physics) in biological systems, an idea emphasized by Al-Khalili (e.g^29^. and films), in a DNA + environment combined with non-equilibrium thermodynamics and chaos theory may lead to collective behavior and an emergent entity leading from chaos to life.

Our recent^5^ and current experiments yielding pH = 8.3(1) are consistent with a pH of 8.2–8.1 that was present in both the alkaline pH of contemporary oceans (Smithsonian, National Institute of Natural History) and the hypothetical pH of the ancient ocean dating back to 3 billion years ago. This finding suggested that this specific pH, along with primary energy accumulation and release processes in DNA molecules, could have played a crucial role in the emergence of life on Earth.

It is important to note that the ideal temperature for the growth of barley is around 20 °C, which is comparable to the temperature of 20.3(1) ^o^C observed, e.g., in the EMF peak of our research. With climate change threatening barley yield, it may be necessary to develop new tools and cultivars to adjust the optimum growth temperature of barley (and other crops) to suit the changing environmental conditions. Our research results could be a starting point in this direction, especially as they can be obtained through inexpensive and transparent investigations.

### DNA stores and releases primary energy

The fields of physics involve, among others, two processes: Maxwell’s demon and Majorana’s fermion, the latter also recognized in superconducting systems as the Bogoliubov quasiparticle. These processes could recreate Bergson’s concept of the “élan vital” (vital force or energy) and temperature-driven entropy oscillations in DNA. On the other hand, DNA emits light, the most affordable form of energy that is both massless and clean, but its role has not yet been determined. Hence, a question arises: why does DNA emit light? The answer is that the light emitted by DNA may be used to transmit energy. This, in turn, allows for the simultaneous (synchronous) transmission of information within the immediate (nearest) neighborhood of DNA molecules which can supposedly regulate the coherent development and functioning of living organisms. It could also trigger a signaling cascade that initiates growth in the DNA world in response to favorable environmental conditions.

### Perspective

The light produced by DNA molecules has immense potential in theoretical research and the practical development of DNA-based molecular electronics. This could ultimately lead to the development of a molecular hybrid computer (DNA quantum computer) capable of operating at room temperature. It’s astounding to think that DNA molecules hold the key to unlocking the potential of quantum computing. By constructing a hybrid quantum computer that merges quantum calculation with classical error correction^5^, we could be on the verge of a computing revolution that could solve previously unsolvable optimization problems.

## Materials and methods

### DNA extraction

Genomic DNA was isolated from the leaf tissue of barley (*Hordeum vulgare*) plants of the cultivar Sebastian. The plants were grown in a growth chamber under a 16/8 h photoperiod at 20 °C. Leave samples were collected in silica gel to remove water from the tissue. The dried tissue was ground in a single step using an electric mill (Retsch, MM200, and MM301) and 3 mm glass balls (Sigma‒Aldrich). A modified micro-CTAB method was used for DNA extraction^35^. DNA quantity and quality were measured using a NanoDrop™ ND-1000 UV‒Vis Spectrophotometer. DNA was diluted to 100 ng/µl using TE buffer containing 10 mM TrisHCl and 1 mM EDTA (pH 8.3).

### Staining

DNA was stained with 2% acetocarmine (4 µl acetocarmine solution / 100 µl of DNA).

### Measuring device

The measuring instrument used in the experiment comprised an external polystyrene thermostat (21 × 15 × 14 cm) that was covered with black cardboard to prevent the entry of any external light. The internal measuring chamber was made of thermally insulated polystyrene (638 cm^3^) and was coated with an aluminum-containing semiconductor-solute interface^6^. A photovoltaic semiconductor plate, also known as a detector or cell (Figure S3), was used, which had an n-p junction on a phosphorus-boron Si crystal^14^. A photovoltaic cell converts light energy into electrical energy through the photovoltaic effect. This is the generation of electric current when a semiconductor material is exposed to light. The humidity level during the experiments was around 23%, achieved through water evaporation from a cotton cosmetic flake.

### Electromotive force measurements

To measure the electromotive force (EMF), we transferred 50 µl of the barley genomic DNA sample onto a photovoltaic semiconductor detector (generating approximately 0.5 V when illuminated by sunlight). We conducted the 92 measurements in an internal dark chamber for 15 min at 4.1 Hz while maintaining the external temperature between 19.0 and 24.0 °C. The measuring chamber was kept at a constant temperature or cooled with ice water to prevent energy input. We took DC voltage ($\:U$) measurements (digital filter on) in the physiological temperature range. We captured the mean field of the collective of the DNA strands using a DMM 4040 6–1/2 digit precision multimeter from Tektronix, Inc. We recorded the data as a 15-minute time series (4000 points) on external media (pen drive). Usually, we considered each period, but in some cases where the experimental conditions were not preserved (i.e. the temperature error exceeded +/- 0.05 °C), we rejected the experiments. We were able to make these measurements because of subtle effects, such as the bending of the energy bands, that occurred at the interface between the semiconductor and the liquid (Schmickler and Santos, 2010). On the other hand, the photovoltaic (n-p) detector plate (Figures S3 and S4) could detect light quanta (photons) emitted by the DNA sample. These particles have an energy level equal to the energy measured in the experiment. 

### Current measurements

The current ($\:I$) measurements (digital filter on) were taken in the physiological temperature range; a mean field of the collective of the DNA strands was captured using a DMM 4040 6–1/2 digit precision multimeter from Tektronix, Inc., and the data were subsequently recorded as a 15-min time series on external media.

### Data analysis

The noninvasive solute-semiconductor interface technique collected time series data (*N* = 4000) at different temperatures. After detrending, the data was analyzed using a program written in the R programming language. The measurements were taken at a sampling frequency of 4.1 Hz. They included the electromotive force (EMF) in volts (V) and the generated photovoltaic current $\:I$ in amperes (A) as a function of time at each temperature. The EMF, electrical current, and dynamic entropy^36^ (calculated using the approximated entropy procedure in the R programming language) were plotted for a narrow range of physiological temperatures to study their behavior. The Hurst exponent was used to measure the long-term memory of time series data. The Hurst exponent, denoted as $\:H$, relates to the autocorrelations of a time series and the rate at which these correlations decrease as the lag between pairs of values increases. It’s often called the “index of dependence” or “index of long-range dependence”. For self-similar time series, the Hurst exponent is directly related to the fractal dimension ($\:D$): $\:D=2-H$. Higher $\:H$ values indicate a smoother trend, less volatility, and less roughness in the data. In this work, it was calculated using the R procedure.

## Electronic supplementary material

Below is the link to the electronic supplementary material.

Supplementary Material 1

Supplementary Material 2

## Data Availability

The datasets used and/or analysed during the current study available from the corresponding author on reasonable request.
