# Supplementary material for: Ultra-weak photon emission from DNA
Source: Sci Rep. 2024 Nov 21;14:28915. doi: 10.1038/s41598-024-80469-0 (PMC11582580; doi:10.1038/s41598-024-80469-0)
Supplement: Supplementary file 2 — Supplementary Material 2 [file 41598_2024_80469_MOESM2_ESM.docx]

SUPPLEMENTARY INFORMATION

Ultra-weak photon emission from DNA

Mariusz Pietruszka and Marek Marzec

*University of Silesia, Faculty of Natural Sciences, Institute of Biology, Biotechnology, and Environmental Protection; 40-032 Katowice, Poland*


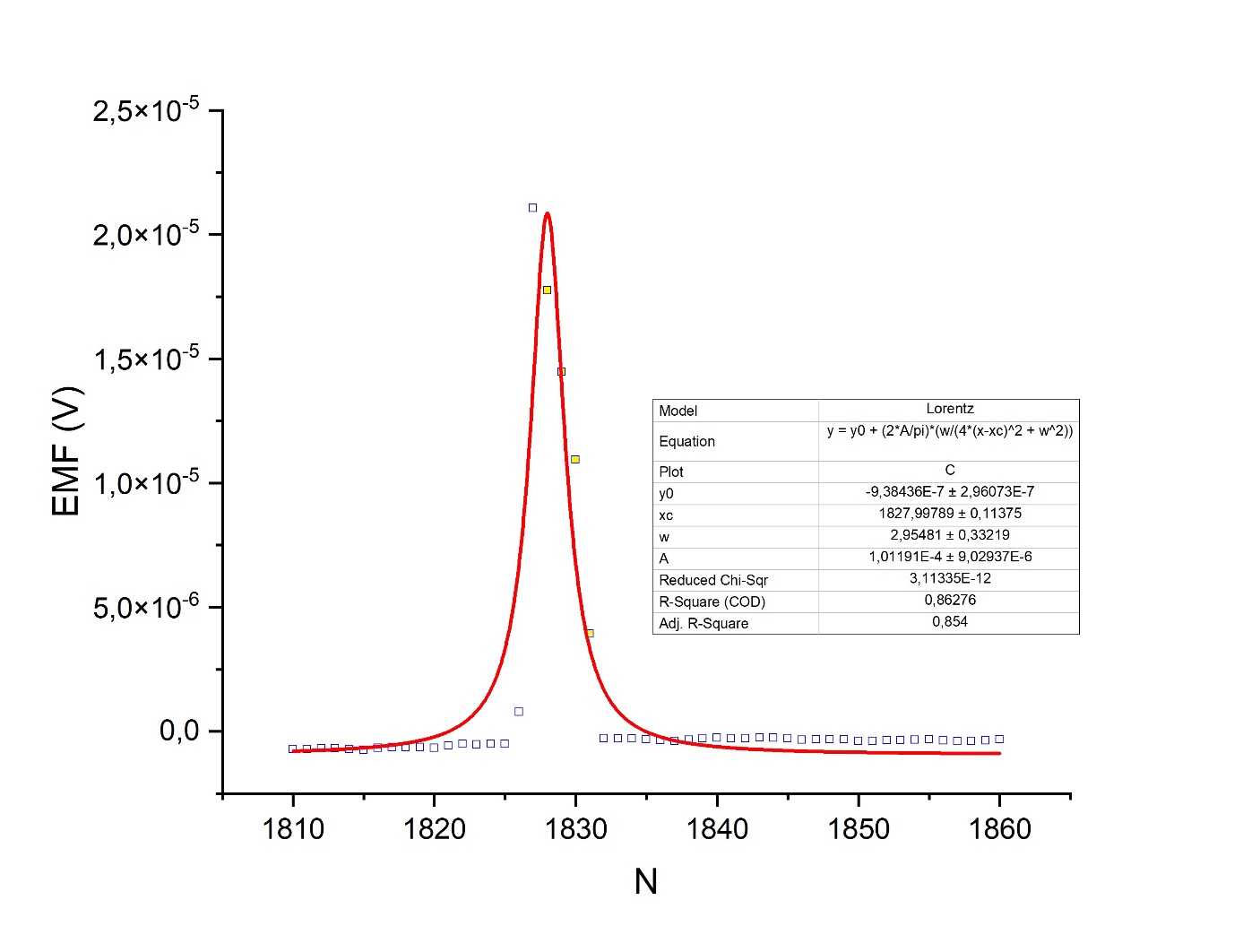


**Figure S1.** The resonant peak of electromotive force (EMF) is interpolated by Lorentz distribution; peak asymmetry and relaxation (approx. 1 s) show the excited state decay. The plot can be considered *a demonstration of a sensed photon* (compare with a red curve in Fig. 5 in “Generating Single Photons on Demand” by Vladan Vuletic, MIT Physics Annual 2005), emitted by DNA.

^
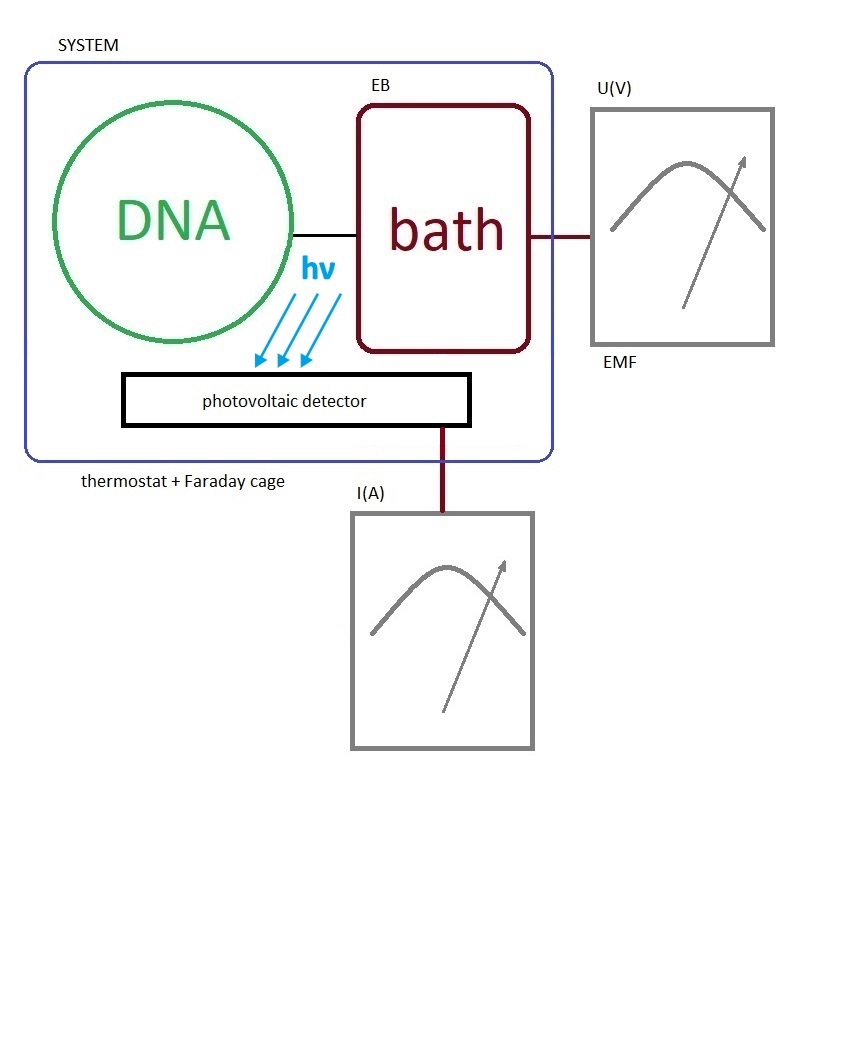
^

**Figure S2.** Schematics of the setup: the system (DNA+TE) interacts with a bath (TE) and is continuously monitored by their respective measurement apparatus.


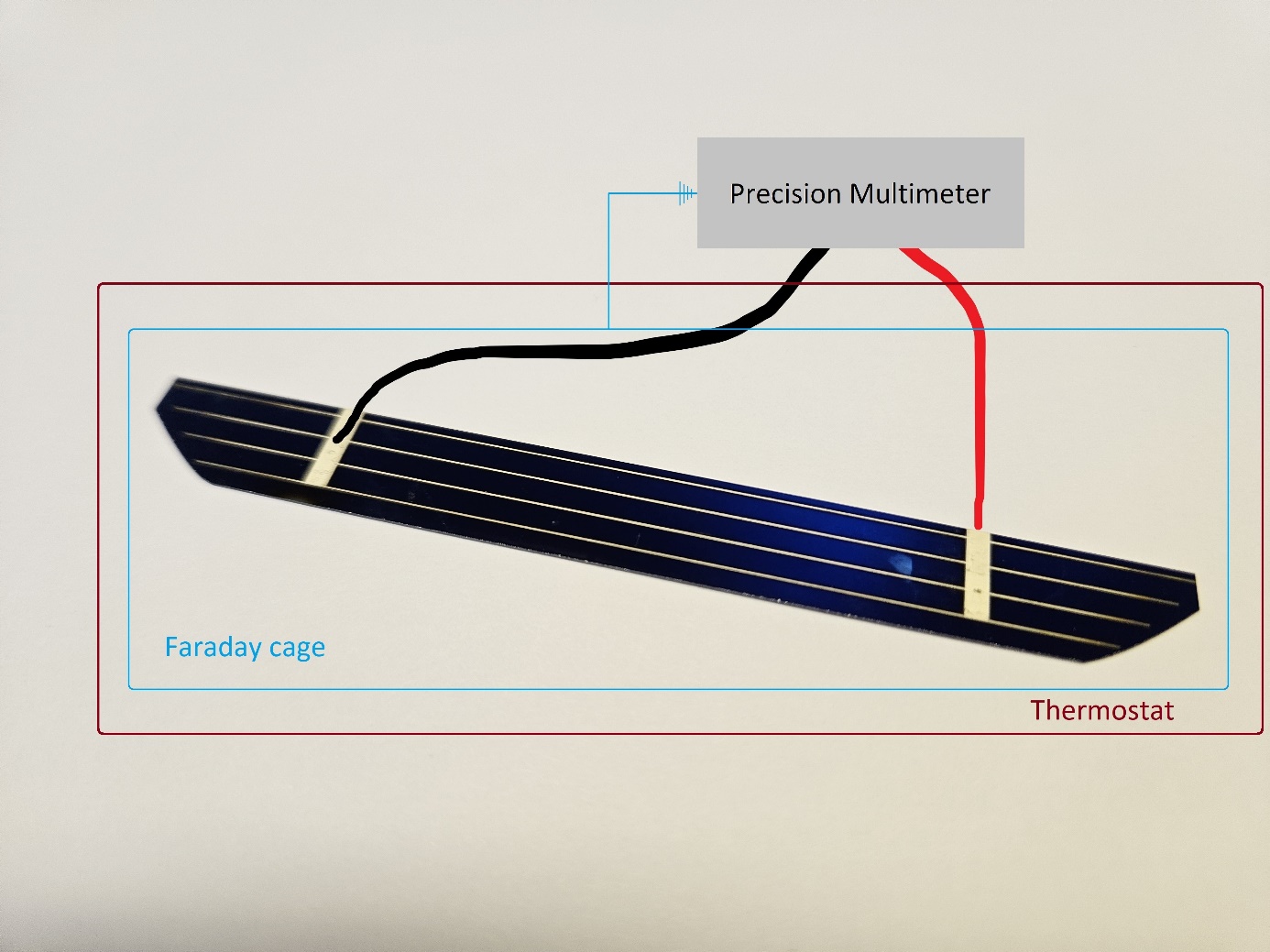


**Figure S3.** The instrument’s photovoltaic detector. Red and black wires – the screened copper wires. Note, that the leads are attached to opposite sides.


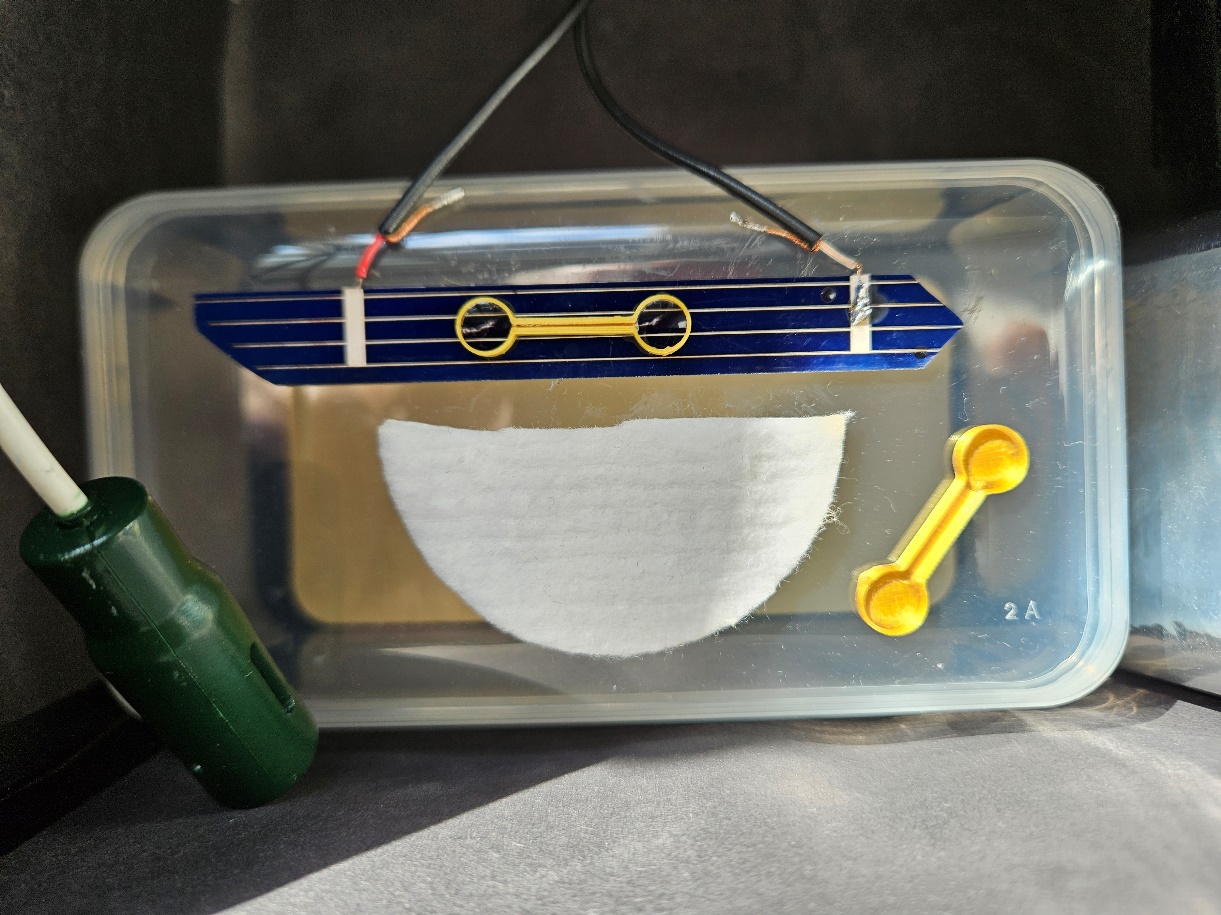


**Figure S4.** A photovoltaic detector and a 3D-printed double-dot compartment and lid (yellow) are used in a double-dot experiment. A temperature sensor (green) is also used, but the aluminum Faraday cage is not shown. The state of the contact slit depends on the type of experiment being conducted and it can either be closed or open.

**
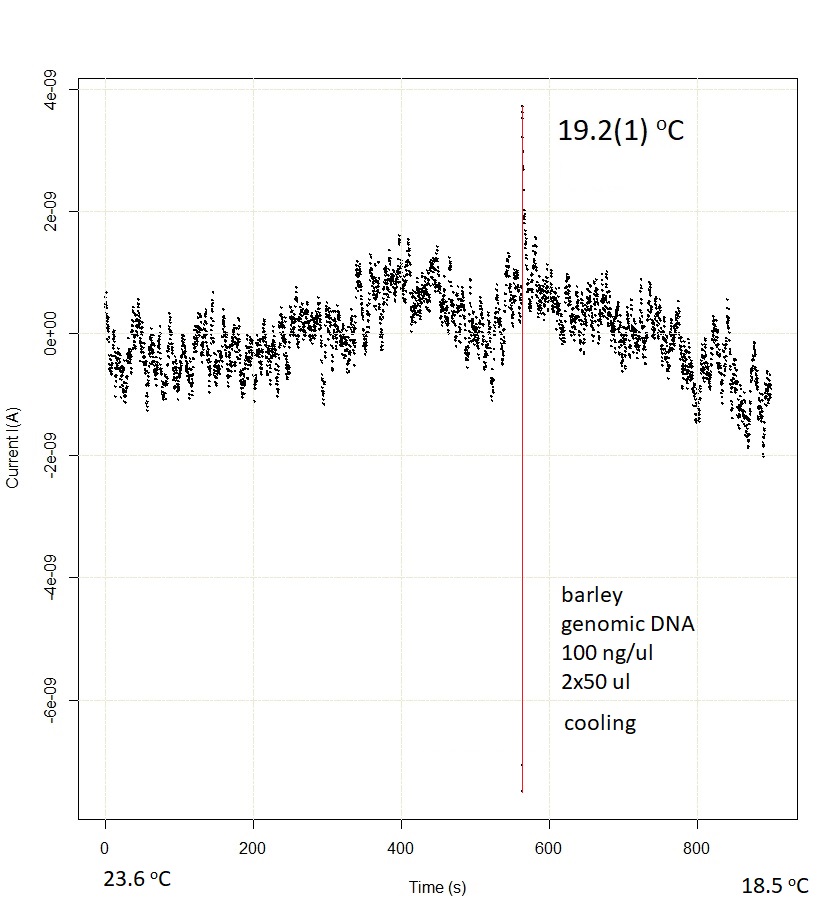
**

**Figure S5.** Electric current (I) as a function of time/temperature (detrended data). A sample was cooled using melting ice to record temperature-dependent data without adding heat to the system. The stained sample caused, due to ion-intercalation processes, a noisy pattern compared to Figure 5, but the main peak (dotted red line) still occurred.

**
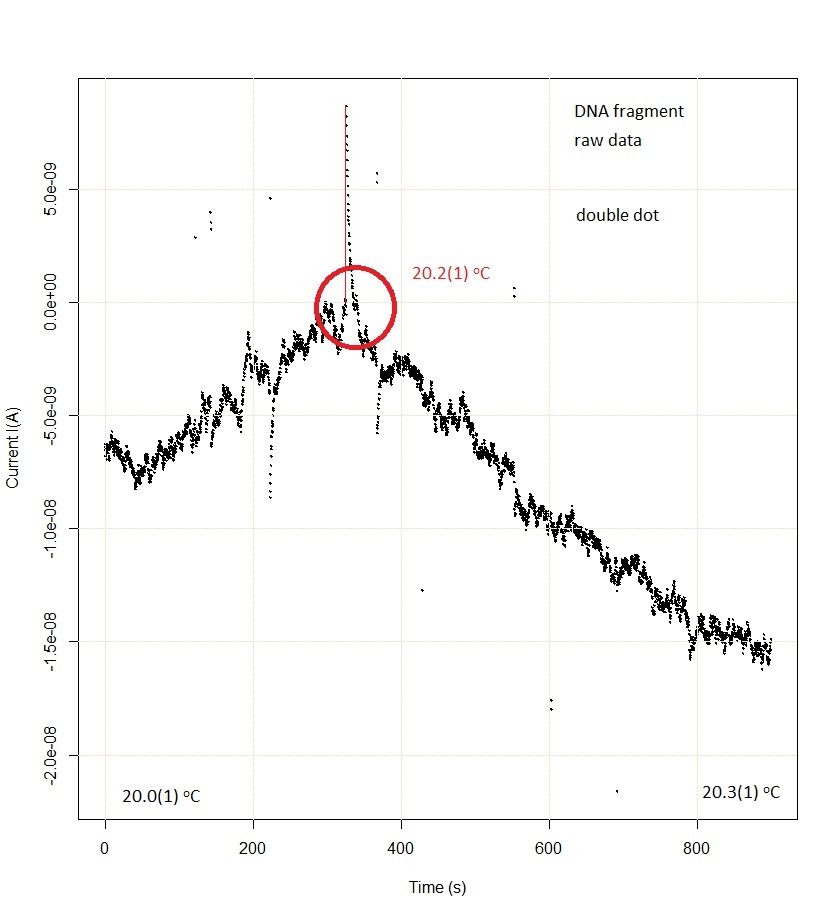
**

**Figure S6.** A current peak occurs at zero bias voltage in a double-dot experiment (raw data). Two absorption peaks and one emission peak (indicated) appear at about 20.2(1) ^o^C.

**
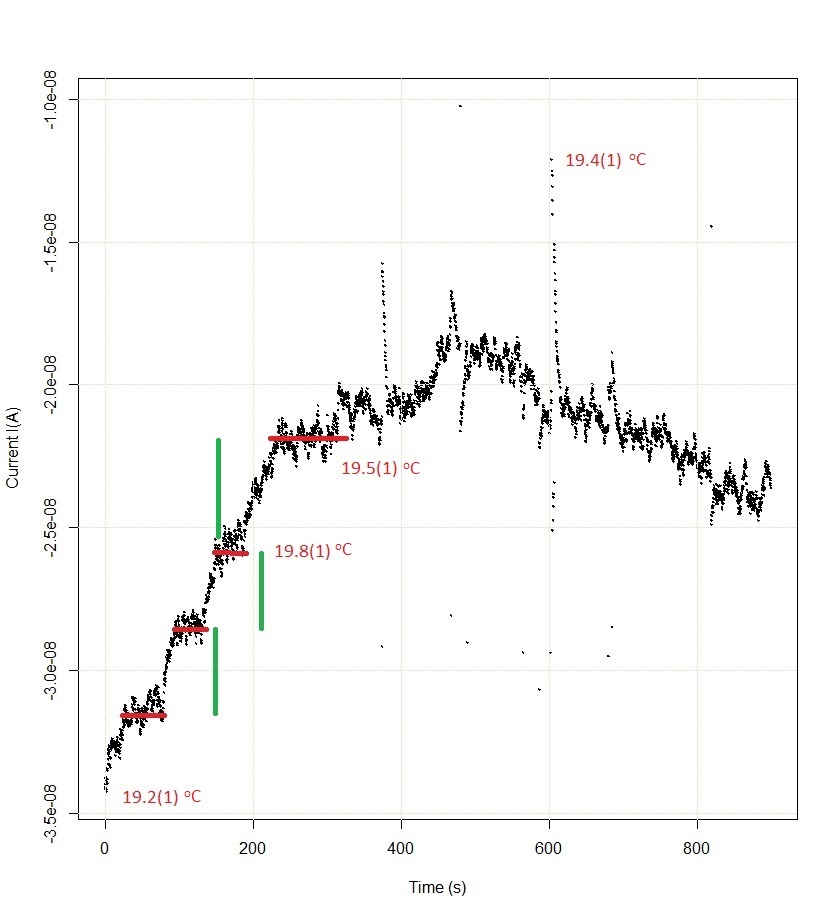
**

**Figure S7.** The quantized levels in DNA. Steps in a current occur at zero bias voltage in a double-dot experiment (raw data, closed bridge). The corresponding temperatures are indicated in the plot.

**
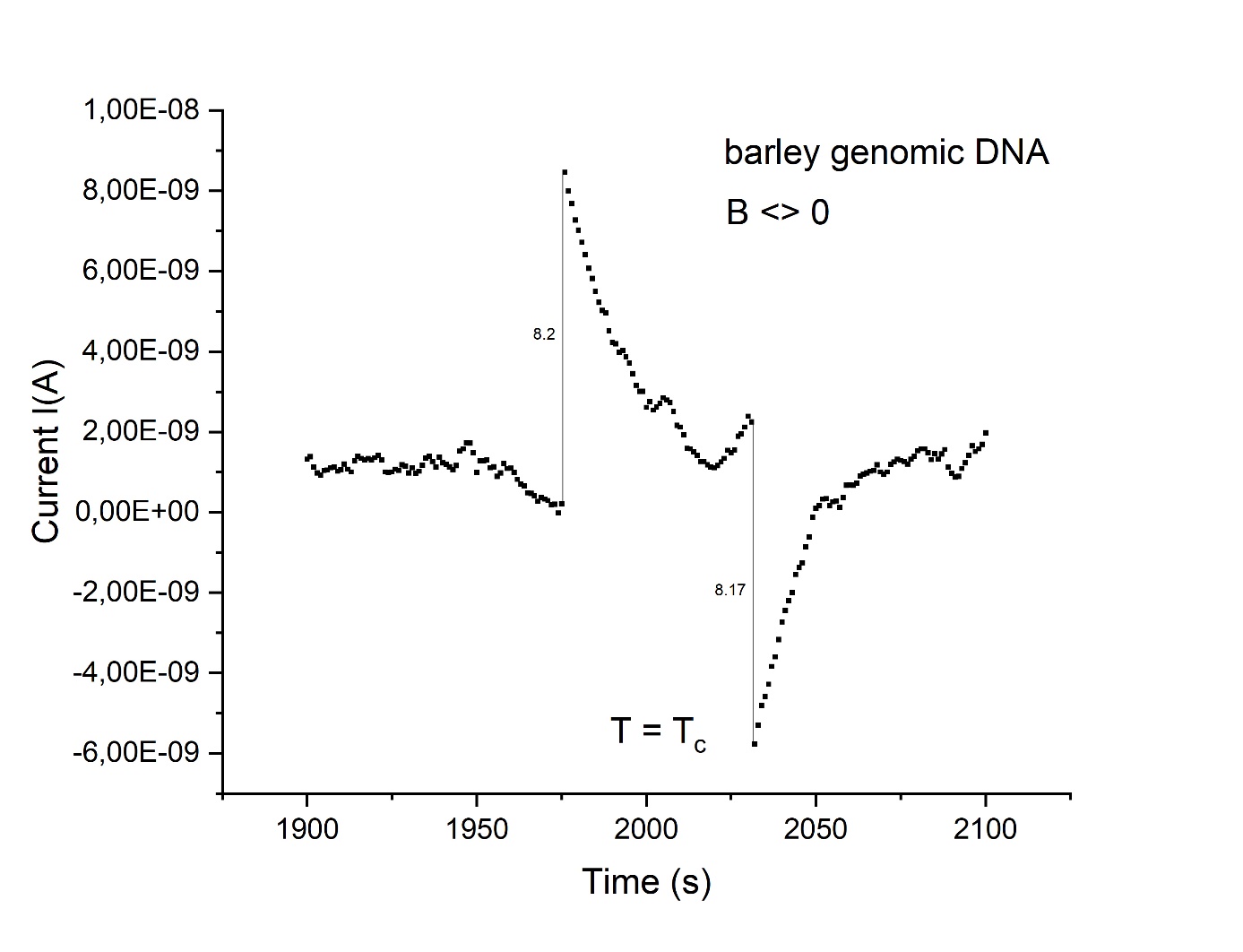
**

**Figure S8.** The magnified transition point of the one presented in Figure 9, represents a photon emission/absorption process. The almost equal lengths of a jump (x 1E-08) are indicated in the plot. The value of Tc is approximately 20.2°C (295.35 K), corresponding to 0.02545 eV.

**
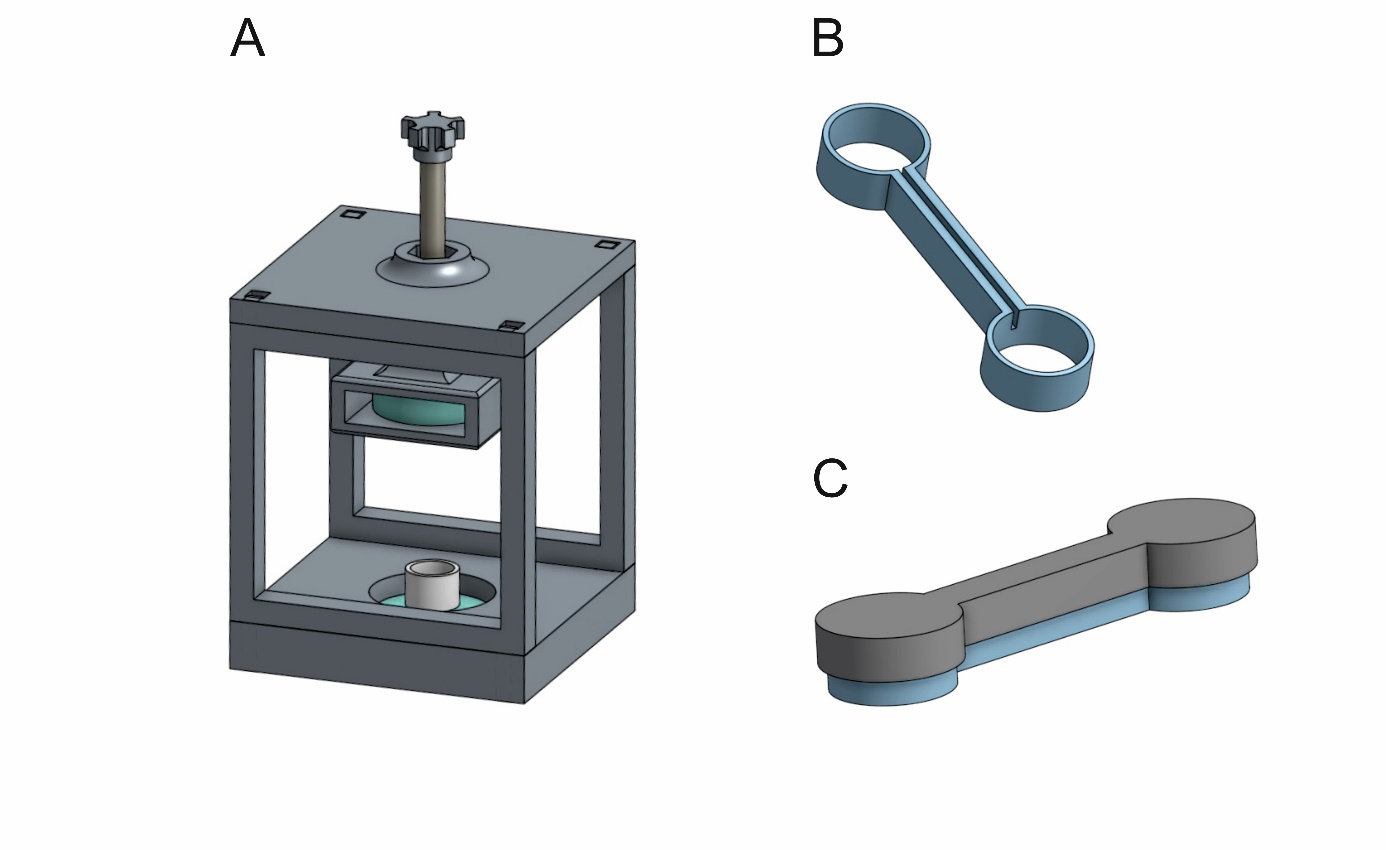
**

**Figure S9.** The experimental setup for measuring magnetic fields involves two neodymium magnets (A) placed above and below the containers (B), with the system covered for humidity stabilization (C). Courtesy of Wojciech Brzęk (3D printing).

**Movie 1.** The Tektronix DMM 4040 6-1/2 Digit Precision Multimeter displays visible current oscillations in a several-second recording.
